# Immune profiling of pre- and post-treatment breast cancer tissues from the SWOG S0800 neoadjuvant trial

**DOI:** 10.1186/s40425-019-0563-7

**Published:** 2019-04-10

**Authors:** Xiaotong Li, Sarah Warren, Vasiliki Pelekanou, Vikram Wali, Alessandra Cesano, Mingdong Liu, Patrick Danaher, Nathane Elliott, Zeina A. Nahleh, Daniel F. Hayes, Gabriel N. Hortobagyi, William E. Barlow, Christos Hatzis, Lajos Pusztai

**Affiliations:** 1 Yale Cancer Center, Breast Medical Oncology, Yale School of Medicine, 300 George Street, Suite 120, Rm133, New Haven, CT 06511 USA; 2 NanoString Technologies Inc., Seattle, WA USA; 3 Department of Pathology, Yale School of Medicine, New Haven, CT USA; 4 Cleveland Clinic Florida, Maroone Cancer Center, Weston, FL USA; 5 University of Michigan Rogel Cancer Center, Ann Arbor, MI USA; 6 University of Texas MD Anderson Cancer Center, Houston, TX USA; 7 SWOG Statistical Center, Seattle, WA USA

**Keywords:** Tumor infiltrating lymphocytes, PD-L1, Immune-related genes, Neoadjuvant treatment, Bevacizumab

## Abstract

**Background:**

How the immune microenvironment changes during neoadjuvant chemotherapy of primary breast cancer is not well understood.

**Methods:**

We analyzed pre- and post-treatment samples from 60 patients using the NanoString PanCancer IO360™ assay to measure the expression of 750 immune-related genes corresponding to 14 immune cell types and various immune functions, and assessed TIL counts and PD-L1 protein expression by immunohistochemistry. Treatment associated changes in gene expression levels were compared using t-test with Bonferroni correction. TIL count, PD-L1 protein and immune metagenes were compared using Wilcoxon test. Baseline immune markers were correlated with pathologic complete response (pCR) using estrogen receptor and treatment arm adjusted logistic regression.

**Results:**

At baseline, high TIL counts and high expression of chemoattractant cytokines (CCL21, CCL19) and cytotoxic T cell markers were associated with higher pCR rate. High expression of stromal genes (VEGFB, TGFB3, PDGFB, FGFR1, IGFR1), mast and myeloid inflammatory cell metagenes, stem cell related genes (CD90, WNT11, CTNNB1) and CX3CR1, and IL11RA were associated with residual disease (RD). After treatment, in cases with pCR, TIL counts and most immune genes decreased significantly. Among RD cases, TIL counts and PD-L1 expression did not change but cellular stress and hypoxia associated genes (DUSP1, EGR1), and IL6, CD36, CXCL2, CD69 and the IL8/VEGF metagene increased.

**Conclusions:**

Activated T cells in the tumor microenvironment are associated with pCR whereas stromal functions are associated with residual disease. Most immune functions decrease during neoadjuvant chemotherapy but several immunotherapy targets (PD-L1, IL6, IL8) remain expressed in RD suggesting potential therapeutic strategies.

**Electronic supplementary material:**

The online version of this article (10.1186/s40425-019-0563-7) contains supplementary material, which is available to authorized users.

## Background

Immune cells in the microenvironment of breast cancer influence, and may partially mediate, response to chemotherapy [1–3]. Pre-operative (neoadjuvant) chemotherapy of newly diagnosed, early stage breast cancer where pre- and post-treatment tissues are available provides an opportunity to study baseline immune parameters that are associated with treatment response and to assess changes in the immune microenvironment caused by treatment. Several studies have reported that the higher the tumor infiltrating lymphocyte (TIL) count, or immune-related gene expression at baseline, the greater the probability of pathologic complete response (pCR) to preoperative chemotherapy in all breast cancer subtypes [3–6]. However, the contribution of various lymphocyte and immune cell sub-types to determining treatment response remains unknown. To date, few studies examined immunological changes in the breast tumor microenvironment after chemotherapy [7, 8]. Better understanding of the immune functions that influence the probability of pathologic complete response (pCR) to preoperative chemotherapy could suggest novel immunotherapeutic strategies to enhance chemotherapy efficacy. Patients with extensive residual invasive cancer in the breast or lymph nodes after preoperative chemotherapy have guarded prognosis, particularly if they have triple negative breast cancer [9]. Better understanding of the immune milieu of residual cancers may also suggest novel therapeutic approaches to improve the prognosis of these patients.

S0800 (NCT00856492) was a randomized, 3-arm, Phase II trial that tested if inclusion of bevacizumab with neoadjuvant chemotherapy could improve pCR rates in HER2-negative, locally advanced, or inflammatory breast cancer (IBC). The three arms of the trial were weekly nab-paclitaxel and bevacizumab followed by dose-dense doxorubicin/cyclophosphamide (ddAC) (Arm A), nab-paclitaxel followed by ddAC, (Arm B), and ddAC followed by nab-paclitaxel (Arm C). Patients were randomly allocated in 2:1:1 ratio to arms A, B and C, respectively. For the primary efficacy analysis, the arms B and C were combined. The trial demonstrated that bevacizumab increased pCR rate from 21 to 36% (*p* = 0.019) but chemotherapy sequence in the non-bevacizumab arms did not significantly influence efficacy [10]. The main objectives of the current study were to (i) examine the association between pCR and pre-treatment TIL count, PD-L1 protein expression and expression of 750 immune-related genes and (ii) assess changes in these immune parameters in paired pre- and post-treatment tissues.

## Methods

### Patients and samples

Of the 215 patients accrued to the S0800 trial, 134 patients had pre-treatment and 63 patients had post-treatment formalin fixed paraffin embedded (FFPE) tissues with consent for research, including 60 patients with paired tissues (Additional file 1: Figure S1). Pathologic complete response was determined by the local pathologists and was defined as the absence of any residual invasive cancer in the breast and axilla (pCR = ypT0/is, ypN0). Patients who had any viable residual cancer after chemotherapy, regardless of clinical response, were categorized as residual disease (RD). The current biomarker analysis was approved by the NCI and the Yale Cancer Center Human Investigations Committee. Demographic and disease characteristics for the overall trail and current immune marker study populations are shown in Table 1.

**Table 1:**
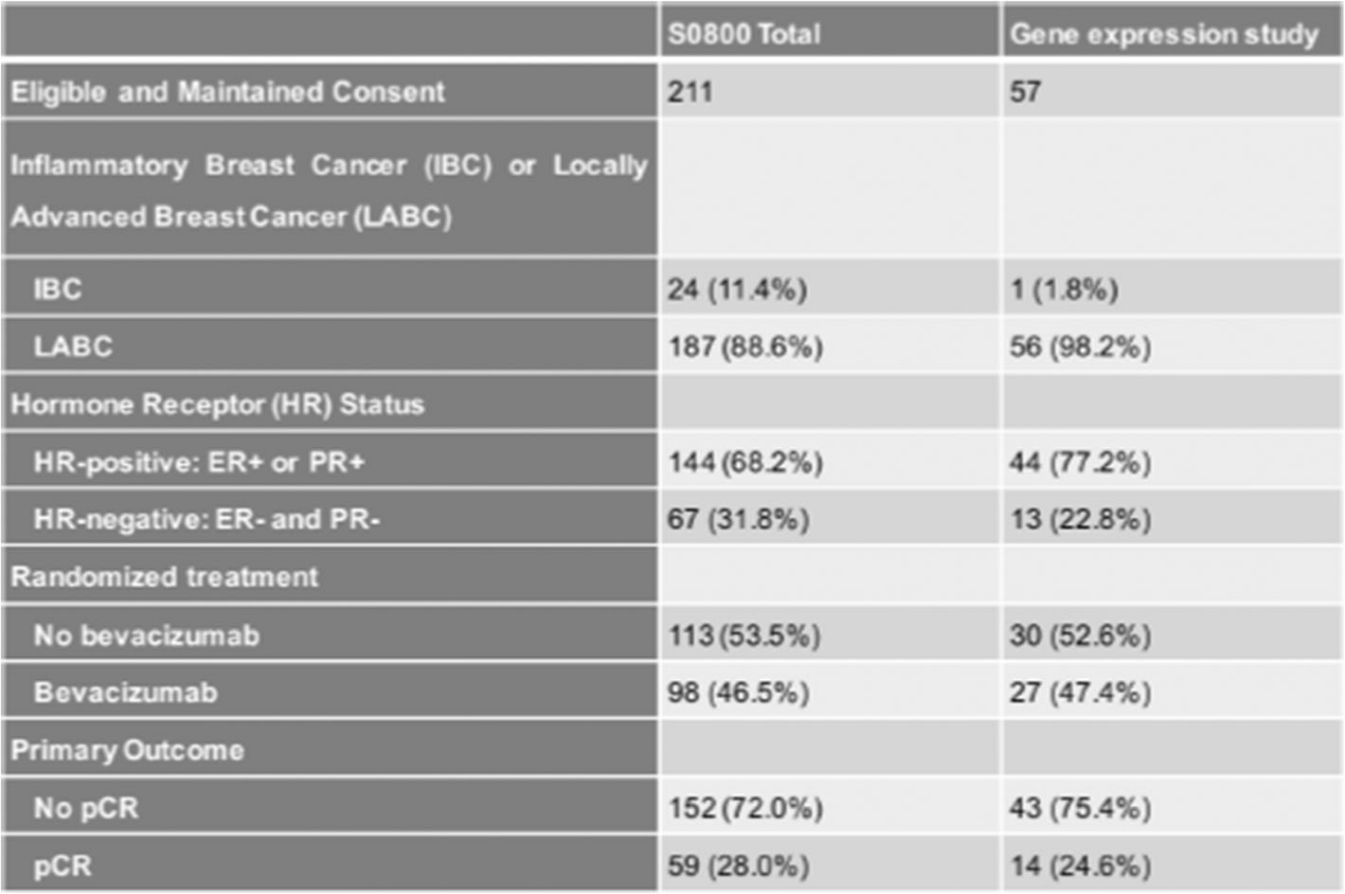
Demographic and disease characteristics for the overall trial population and the immune-related gene expression subset.

### TIL assessment

TILs counts were determined by a pathologist (V.P.) on hematoxylin eosin stained full sections following the scoring guidelines of the International Immuno-Oncology Biomarker Working Group on Breast Cancer [11]. Stromal TIL scores were defined as the percentage of tumor stroma area that was occupied by mononuclear inflammatory cells. Inflammatory infiltrates in the stroma of noninvasive lesions and normal breast structures were excluded from TIL counts. In cases with pCR, the original tumor bed was examined and scored.

### PD-L1 immunohistochemistry

PD-L1 immunohistochemistry (IHC) was performed on 5 μm FFPE whole tissue sections using the FDA cleared 22C3 assay on the Dako Link 48 staining platform following the manufacturer’s instructions [8]. For controls, we used slides from the DAKO 22c3 pharmDx assay that includes a PD-L1 positive (NCI-H226) and a PD-L1 negative (MCF-7) cell line. Staining in tumor and stromal cell compartments were scored separately as a percentage of cells with PD-L1 signal of any intensity. PD-L1 positivity was defined as > 1% of tumor or stromal cells staining positive. This threshold was used in metastatic breast cancer trials for patient selection for anti-PD-1 therapy with pembrolizumab and atezolizumab [12].

### Transcriptional profiling

Total RNA was isolated from 5 μm FFPE sections using the Qiagen RNeasy FFPE kit and 100 ng RNA was hybridized to the beta version of the NanoString PanCancer IO 360 code set and read on the nCounter platform (NanoString). The expression 750 immune-related genes and 20 housekeeping genes were assessed. The nSolver 2.6 software was used to normalize expression values using housekeeping genes following the manufacturer’s recommendations [13]. Twenty of the 120 samples were excluded from further analysis due to low geometric mean of housekeeper gene expression. The remaining 100 samples from 57 patients included 43 pairs of matched pre- and post-treatment samples. Genes were grouped into 14 immune cell type metagenes (total T, Th1, Treg, Total CD8, exhausted CD8, Cytotoxic T, B, NK, NK-CD56, Mastoid cell, CD45, Dendritic cell, macrophage, neutrophil) and 39 immune functions according to the manufacturer’s designation (Additional file 2: Table S1). The metagene scores were calculated as the geometric mean expression of the member genes [14]. We also examined 26 previously published prognostic and immune therapy response predictive metagenes (Additional file 3: Table S2). We calculated these metagene scores following the methods described in the respective publications. Normalized gene expression data is deposited in the Gene Expression Omnibus database (GSE114403).

### Statistical analysis

All immune marker data was generated without knowledge of the patient outcomes. TIL counts and PD-L1 expression levels were compared between the pCR and RD groups using the Wilcoxon rank-sum test and between the pre- and post-treatment samples using the paired Wilcoxon test. Baseline immune gene mRNA expression was correlated with pCR using estrogen receptor (ER) and treatment arm adjusted logistic regression. Treatment associated changes in gene expression levels were compared using t-test with Bonferroni correction.

## Results

### TIL counts

**Fig. 1:**
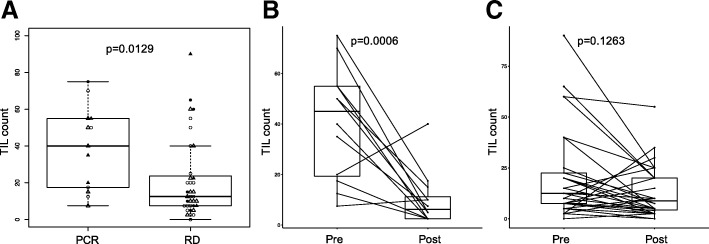
Tumor infiltrating lymphocyte (TIL) counts in cases with pathologic complete response (pCR) and residual disease (RD), and in pre- and post-treatment samples. **a** Box plots of baseline TIL counts in cases with pCR and RD. Triangles and dots/circles represent patients in the bevacizumab and control arms, respectively. Open and dark symbols indicate estrogen receptor positive and negative patients, respectively. **b** Changes in TIL counts in the tumor bed of cases with pCR. **c** Changes in TIL counts in cases with RD. Paired pre- and post-treatment samples are connected by lines to indicate up or down change in each individual. *P* values are from Wilcoxon test

At baseline, cases with pCR had significantly higher TIL counts than cases with RD (*p* = 0.0129, Fig. 1a). TIL counts remained significantly associated with pCR (*p* = 0.0243), after adjustment for ER status and treatment arm.

Among patients with pCR, TIL counts were significantly lower in post-treatment samples compared to pre-treatment (*p* = 0.00063, Fig. 1b). Among RD cases, there was no significant difference in pre- and post-treatment TIL counts (*p* = 0.1263, Fig. 1c). However, a substantial subset of cases had numerically decreased post-treatment TIL count (*n* = 20) while a in a few cases TIL count increased (*n* = 8), but there was no difference in disease-free or overall survival by decrease or increase in TIL count in the residual cancer.

### PD-L1 protein expression in tumor and stromal cells

**Fig. 2:**
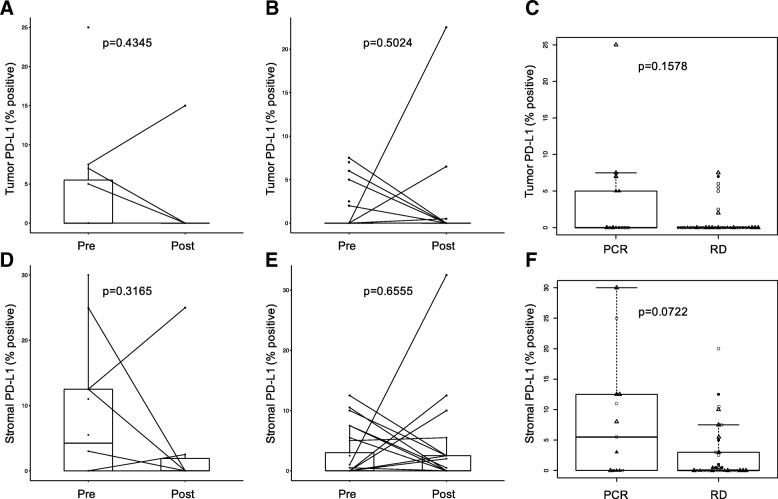
PD-L1 protein expression in cases with pathologic complete response (pCR) and residual disease (RD), and in pre- and post-treatment samples. **a** PD-L1 expression changes on tumor cells in cases with pCR (Wilcoxon test *p* = 0.435). **b** PD-L1 expression changes on tumor cells in cases with RD (Wilcoxon test *p* = 0.502). Pre- and post-treatment samples from the same patient are connected with lines. **c** PD-L1 expression comparisons between pCR and RD cases for all baseline samples (Wilcoxon test *p* = 0.1578). Shape and color of each dot represent treatment arm and ER status. **d**-**f** Similar figures as **a**-**c** showing changes and PD-L1 changes in stromal cells. Symbols ae the same as on Fig. 1

Tumor cell PD-L1 protein expression did not change significantly after treatment, in either response group (Fig. 2a, b) and there was no association between baseline PD-L1 expression and response to therapy (Fig. 2c). Stromal PD-L1 expression was also similar between pre- and post-treatment samples (Fig. 2d, e) and between response groups (Fig. 2f).

**Fig. 3:**
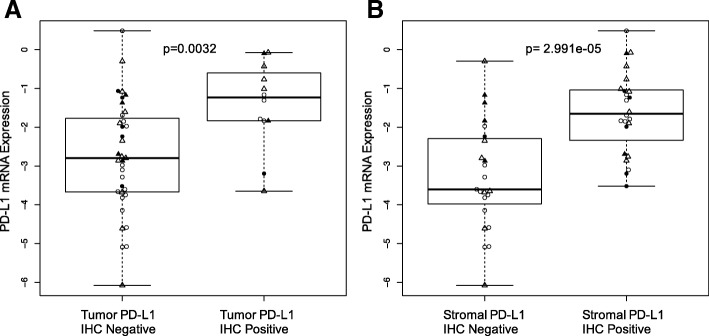
PD-L1 protein and mRNA expression. **a** PD-L1 mRNA levels in cases with positive and negative PD-L1expression on tumor cells. **b** PD-L1 mRNA levels in cases with positive and negative PD-L1expression on stromal cells. Symbols are the same as on Fig. 1. P values are from Wilcoxon test

We also compared PD-L1 expression by immunohistochemistry and by mRNA measurements. Samples showing positive PD-L1 protein expression, in either stromal or cancer cells, had significantly higher PD-L1 (CD274) mRNA expressions than samples showing no PD-L1 protein expression (Fig. 3).

PD-L1 percent positivity either on tumor (r^2^ = 0.23, *p* = 0.04) or stromal cells (r^2^ = 0.38, *p* = 0.0003) correlated weakly but significantly with TIL percent count (Additional file 4: Figure S4 A, B). PD-L1 positive cases, either on tumor or stromal cells, also showed significantly higher TIL counts compared to PD-L1 IHC negative cancers (Additional file 4: Figure S4 C, D).

### Baseline immune gene expression and response to therapy

**Fig. 4:**
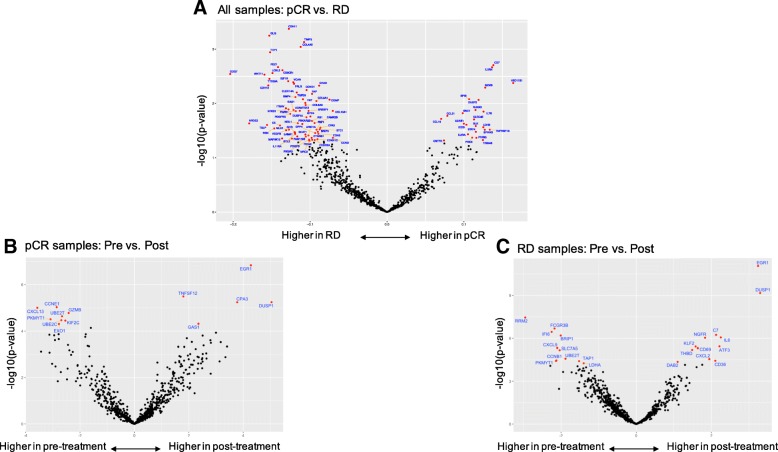
Immune genes associated with response and immune gene expression changes after treatment in cases with pathologic complete response (pCR) and residual disease (RD), respectively. **a** Volcano plots of the log odds ratio of gene expression by pathologic response status. Each dot represents a gene, the x-axis shows the log2-transformed correlation coefficient for response. Positive values indicate higher expression in the pCR cohort, negative values indicate higher expression in the RD cohort. The y-axis shows -log10-transformed *p*-values from logistic regression, genes with *p* < 0.05 are colored red and annotated with names. **b** Volcano plot of gene expression changes in pre- and post-treatment pCR samples. **c** Volcano plot of gene expression changes in pre- and post-treatment RD samples. On panels B and C, the x-axis shows the log2-transformed expression difference between pre- and post-treatment tissues; positive values indicate higher expression in post-, and negative values indicate higher expression in pre-treatment samples. The y-axis shows -log10-transformed p-values. Genes that remained significant after Bonferroni adjustment (adjusted *p* < 0.05) are in red

In ER status and treatment arm adjusted logistic regression analysis, higher expression of 24 immune genes were significantly associated with pCR (Fig. 4a). These included several activated cytotoxic T cell markers such as granzyme, granulysin, CD7 and chemoattractant cytokines CCL21 and CCL19. Expression of IL7R, that promote V(D)J T cell receptor and immunoglobulin recombination during lymphocyte maturation was also higher in cases with pCR.

A different and substantially larger number of immune genes (*n* = 63) were significantly associated with RD (Fig. 4a). Many of these genes were related to fibroblast and stromal functions (COL4A5, COL5A1, COL6A3, COL10A1, TIMP3, CDH-2, − 11, − 13, CHAD, VACN), some representing therapeutic targets including VEGFB, TGFB3, PDGFB/PDGFRB, FGFR1 and IGF1R. VEGF expression was not associated with response to bevacizumab in this study. The low affinity Fc gamma receptor (FCGR2A/CD32), the cytokine receptor CX3CR1 and IL11RA were also higher in cancers with RD. CXCR1 and IL11R represent potential therapeutic targets since they promote macrophage survival and induce apoptosis resistance in cancer cells [14] and also stimulate cancer cell survival and promote angiogenesis and metastasis formation [15], respectively. High expression of the putative cancer and hematopoietic stem cell marker THY1/CD90 and WNT11 and CTNNB1 also suggest potential novel therapeutic strategies to increase pCR rates.

**Fig. 5:**
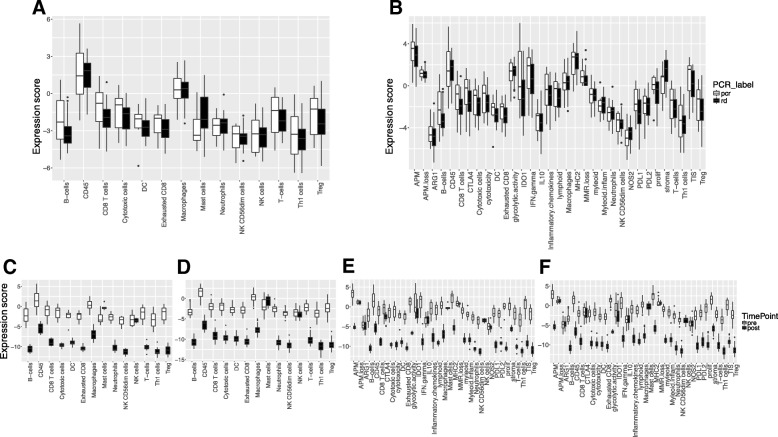
Expression of immune metagenes in cases with pathologic complete response (pCR) and residual disease (RD) and in pre- and post-treatment samples. **a** Expression of immune cell type metagenes in cases with pCR and RD. **b** Expression of immune function metagenes in cases with pCR and RD. **c** Expression of immune cell type metagenes in pre- and post-treatment samples, in cases with pCR. **d** Expression of immune cell type metagenes in pre- and post-treatment samples, in cases with RD. **e** Expression of immune function metagenes in pre- and post-treatment samples in cases with pCR. **f** Expression of immune function metagenes in pre- and post-treatment samples in cases with RD

At the metagene level, most immune cell types and immune functions showed similar or higher baseline expression in cases with pCR compared to RD except a mast cell signature and stroma and myeloid inflammatory cell signatures that were higher in patients with RD (Fig. 5a, b). None of the 26 previously reported prognostic or immunotherapy predictive signatures showed significant association with pCR after adjustment for ER status and treatment arm.

Age was not predictive of pCR in this study (Additional file 5: Figure S2A). MKI67 (Ki67) mRNA expression levels were also similar between cases with RD and pCR at baseline (*p* = 0.107, Additional file 5: Figure S2B) and in the post-treatment samples (*p* = 0.2823, Additional file 5: Figure S2C). Because most cases were stage III invasive ductal carcinomas (*N* = 2 inflammatory breast cancer), we could not correlate clinical stage or histology with pCR.

### Changes in immune gene expression after chemotherapy

At the individual gene level, most immune genes had lower expression in post-treatment samples in both response groups. However, in cases with pCR, cellular stress and hypoxia associated genes (DUSP1, EGR1, CPA3, GAS1) and TNFSF12 showed increased expression post-treatment (Fig. 4b). In cases with RD, DUSP1 and EGR1 also showed increased expression post-treatment as well as IL6, ATF3, CD36, CXCL2, CD69, NGFR, KLF2, THBD, DAB2 and C7 (Fig. 4c). These genes are involved with tissue repair and inflammation after injury. Among known therapeutic targets in clinical development, CTLA4, PD-L1 and IDO1 expression remained unchanged in residual tissues.

At the metagene level, in both response cohorts, all immune cell metagene expressions decreased, except the NK cell metagene that remained the same. The mast cell metagene increased in post-treatment tissues of patients with pCR (Fig. 5c, d). Metagenes representing immune-related functions also decreased in the post-treatment tissues in both response groups except IL10 signaling, NOS2, ARG1 and APM and MMR loss that remained unchanged in both response cohorts (Fig. 5e, f).

**Fig. 6:**
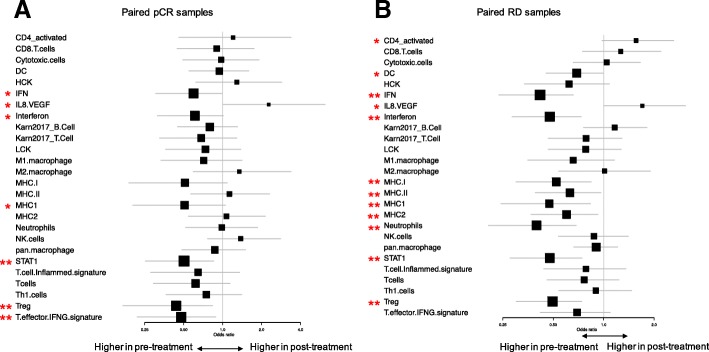
Change in immune metagene and immunotherapy response predictive gene signatures before and after treatment in the two response groups. **a**. Immune gene signature expression changes in pre- and post-treatment samples from pCR cases, represented by odds-ratio and its 95% confidence interval from logistic regression. **b**. Immune gene signature expression changes in pre- and post-treatment samples from RD cases, represented by odds-ratio and its 95% confidence interval from logistic regression. * indicate a trend with 0.05 < *p* < 0.1 and ** indicate significance with *p* < 0.05

We also examined the expression of 26 previously published prognostic and immunotherapy response predictive immune gene signatures. Most of these showed lower expression in post-treatment samples in both response groups (Fig. 6a, b). The decrease was statistically significant for the Interferon, MHC1, STAT1 and Treg signatures in both cohorts. Only the IL8/VEGF gene signature showed significantly higher expression in post-treatment samples in both response groups in (pCR cohort: *p* = 0.038, in RD cohort: *p* = 0.017). The activated-CD4 gene signature also showed significantly higher expression in post-treatment samples of patients with RD (*p* = 0.026).

We have also compared the IL8/VEGF immune gene signature and TIL counts between the treatment arms with or without bevacizumab and found that the IL8/VEGF signature showed significantly higher expression in post-treatment samples in both treatment arms whereas TIL counts were significantly lower in post-treatment samples in both treatment arms (Additional file 6: Figure S3). No treatment-arm specific changes could be detected.

## Discussion

In this study, we examined associations between baseline immune markers and response to nab-paclitaxel and doxorubicin/cyclophosphamide neoadjuvant chemotherapy with or without bevacizumab and assessed changes in the tumor immune microenvironment after treatment. We confirmed earlier observations that higher baseline TIL counts are significantly associated with higher pCR rate even after adjustment for ER and treatment arm [4, 13]. We also identified several immunological functions that provide additional granularity to this association. The significantly higher baseline expression of granzyme and granulysin in the tumor microenvironment of patients who subsequently experience pCR are consistent with the preclinical models that indicate synergy between cytotoxic T cell activity and the antitumor effect of chemotherapy [2, 3]. The high expression of CCL19 and CCL21 in TIL-rich cancers suggests that these chemokines play important roles in attracting T cells, NK cells and mature dendritic cells into the tumor tissues. This raises the possibility re-activation, or supplementation, of these cytokines in immune-poor cancers may turn “immune cold” cancers into “immune hot” tumors. The high baseline expression STAT4, CD7 and IL7R in cases with pCR also suggest that T helper activity and T and B cell maturation are important for achieving pCR whereas the high expression of IL1RN (Interleukin-1 Receptor Antagonist), that suppresses IL1 signaling, suggests that IL-1 activity confers chemotherapy resistance. We were not able to identify any single gene or immune metagene that was selectively predictive of greater sensitivity to bevacizumab. VEGF expression and angiogenesis signatures, had no statistically significant interaction with bevacizumab effect. However, due to the small samples size, the power of this study to detect such interaction is small.

Perhaps more importantly, we also identified a larger number of stroma-related functions and immune parameters that were negatively associated with pCR in all treatment arms. Baseline expressions of mast (MS4A2, CPA3, HDC, TPSB2) and myeloid inflammatory cell metagenes (CXCL1, CXCL3, CXCL2, CCL20, AREG, FOSL1, PTGS2, IER3, IL6) were higher in patients with RD. This raises the possibility that mast cell inhibitors (e.g. masitinib) or IL6 antagonists (e.g. tocilizumab) might convert some patients from RD to pCR. Inhibition of CXCL1, CXCL3, CXCL2, and CCL20 may also increase chemotherapy efficacy. We also noted high baseline expression of several other potential therapeutic targets including VEGFB, TGFB3, PDGFB/PDGFRB, FGFR1 and IGF1R in cancers that did not achieve pCR. The concurrent administration of the anti-VEGF antibody bevacizumab with chemotherapy has increased pCR rates in this and several other randomized neoadjuvant trials (ARTemis, CALGB-40603, SWOG-S0800, NSABP-B40 GeparQuinto), indicating that inhibition of the VEGF axis increases chemotherapy sensitivity. However, two large adjuvant trials (BEATRICE, ECOG-5103) have not shown increased disease-free or overall survival with the inclusion of bevacizumab [16].

When we compared immune parameters in pre- and post-treatment tissues, we noted an almost universal resolution of the immune and inflammatory response in cases with pCR. We previously reported in a larger number of samples from the same study (124 pre- and 62 post-treatment tissues) that TIL counts generally decrease in post-treatment tissues [8]. These observations suggest that either chemotherapy has a cytotoxic effect on TILs or as the size of the primary tumor decreases in response to therapy, the immunogenic target decreases and the corresponding anti-tumor immune reaction also winds down. The greatest decrease in TILs between matched pre−/post-treatment samples is observed in cases with pCR that supports the hypothesis that after complete eradication of the cancer from the breast the immune response also resolves. Indeed, among RD cases, there was no significant difference in pre- and post-treatment TIL counts. However, most immune cell metagene expressions decreased significantly even among cases with RD, except the NK cell metagene that remained the same, and the mast cell metagene that has increased, in post-treatment tissues. The few genes that showed increased expression in residual tissues after pCR included cellular stress and hypoxia induced genes suggesting ongoing tissue repair. In cases with RD, we observed increased expression of a larger number of genes in the residual tissues including several of the same stress- and hypoxia-induced genes (DUSP1, EGR1) as in pCR. The Dual Specificity Phosphatase 1 (DUSP1), is of interest since in some solid tumors it can induce resistance to both chemotherapy and radiotherapy in vitro and in vivo [17]. In addition, we also observed increased expression of IL6 and CXCL2 in RD tissues. Both cytokines were also associated with RD at baseline and represent attractive immuno-oncology targets to enhance chemotherapy efficacy. Several studies implicated high levels of IL6 in poor prognosis and lesser treatment response in breast cancer [18]. The chemokine ligands CXCL-1 and -2 were also shown to promote breast cancer metastasis through myeloid cell recruitment and blocking CXCL-1 and -2 signaling improves chemotherapy efficacy in experimental models [19].

There are a few limitations of this study. Our small sample size prevented us from adequately powered analysis by ER groups, which possess different immunologic characteristics and chemotherapy sensitivities. Several other potentially important subset analyses that we have performed have limited power. We observed no significant difference in survival among residual disease cases that showed increase in TIL count from baseline compared to those that had a decline, we also failed to detect significant association between baseline PD-L1 expression and pCR, as we reported earlier in larger cohorts [5, 8]; these findings most likely reflect underpowered analyses. We also recognize that our pre- and post-treatment sample comparisons may be influenced by unavoidable sampling bias and treatment-related shifts in tumor cellularity. All baseline tissues were from core needle biopsies and all post-treatment samples were from surgically resected tissues and post-treatment samples generally have lower tumor cellularity even in cases with residual disease.

## Conclusions

Our analysis suggests that CCL19, CCL21 and IL7 signaling play an important role in attracting and activating immune cells in the breast cancer microenvironment and might help convert immune cold cancers to immune hot. The presence of activated cytotoxic T cells with granzyme and granulysin expression are important for achieving pCR with chemotherapy. The expression of CXCL1, CXCL3, CXCL2, CCL20, and IL6 are enriched in residual cancer and are associated with lesser response the chemotherapy when highly expressed at baseline. These molecules represent potential novel targets to increase pCR rates and improve outcome in patients with residual cancer after chemotherapy.

## Additional files

Additional file 1: **Figure S1.** CONSORT diagram of samples used in the study. (PDF 11 kb)

Additional file 2: **Table S1.** Gene membership of the immune cell type metagenes and the immune function metagenes. (XLSX 36 kb)

Additional file 3: **Table S2.** Gene membership of the prognostic and immunotherapy response predictive gene signatures and corresponding references. (XLSX 40 kb)

Additional file 4: **Figure S4.** Correlation between TIL counts and PD-L1 expression. A. Correlation between TIL counts and PD-L1 expression on tumor cells. B. Correlation between TIL counts and PD-L1 expression on stromal cells. C. TIL counts in cases with positive and negative PD-L1 expression on tumor cells. D. TIL counts in cases with positive and negative PD-L1 expression on stromal cells. For A and B, Solid line and grey shade represent linear regression line and 95% confidence interval, respectively. Besides, Pearson correlation coefficient r and *p*-value are added. For C and D, *p* values are from Wilcoxon test. (PDF 74 kb)

Additional file 5: **Figure S2.** Age of patient and MKI67 expression in cases with pathologic complete response (pCR) and residual disease (RD). A. Age of patient in cases with pCR and RD. B. MKI67 expressions in all pre-treatment samples with pCR and RD. C. MKI67 expressions in all post-treatment samples with pCR and RD. *P* values are from Wilcoxon test. (PDF 52 kb)

Additional file 6: **Figure S3.** IL8/VEGF signature expression and TIL counts in pre- and post-treatment samples under treatment arms containing or not containing bevacizumab. A. IL8/VEGF signature expression in pre- and post-treatment samples under treatment arm containing bevacizumab. B. IL8/VEGF signature expression in pre- and post-treatment samples under treatment arm not containing bevacizumab. C. TIL counts in pre- and post-treatment samples under treatment arm containing bevacizumab. D. TIL counts in pre- and post-treatment samples under treatment arm not containing bevacizumab. Paired pre- and post-treatment samples are connected by lines to indicate up or down change in each individual. *P* values are from Wilcoxon test. (PDF 58 kb)

## Abbreviations

ddAC: dose-dense doxorubicin/cyclophosphamide
IBC: inflammatory breast cancer
pCR: pathologic complete response
RD: residual disease
TIL: tumor infiltrating lymphocyte
