# Supplementary figures and images for: Immune profiling of pre- and post-treatment breast cancer tissues from the SWOG S0800 neoadjuvant trial
Source: J Immunother Cancer. 2019 Apr 10;7:88. doi: 10.1186/s40425-019-0563-7 (PMC6457012; doi:10.1186/s40425-019-0563-7)

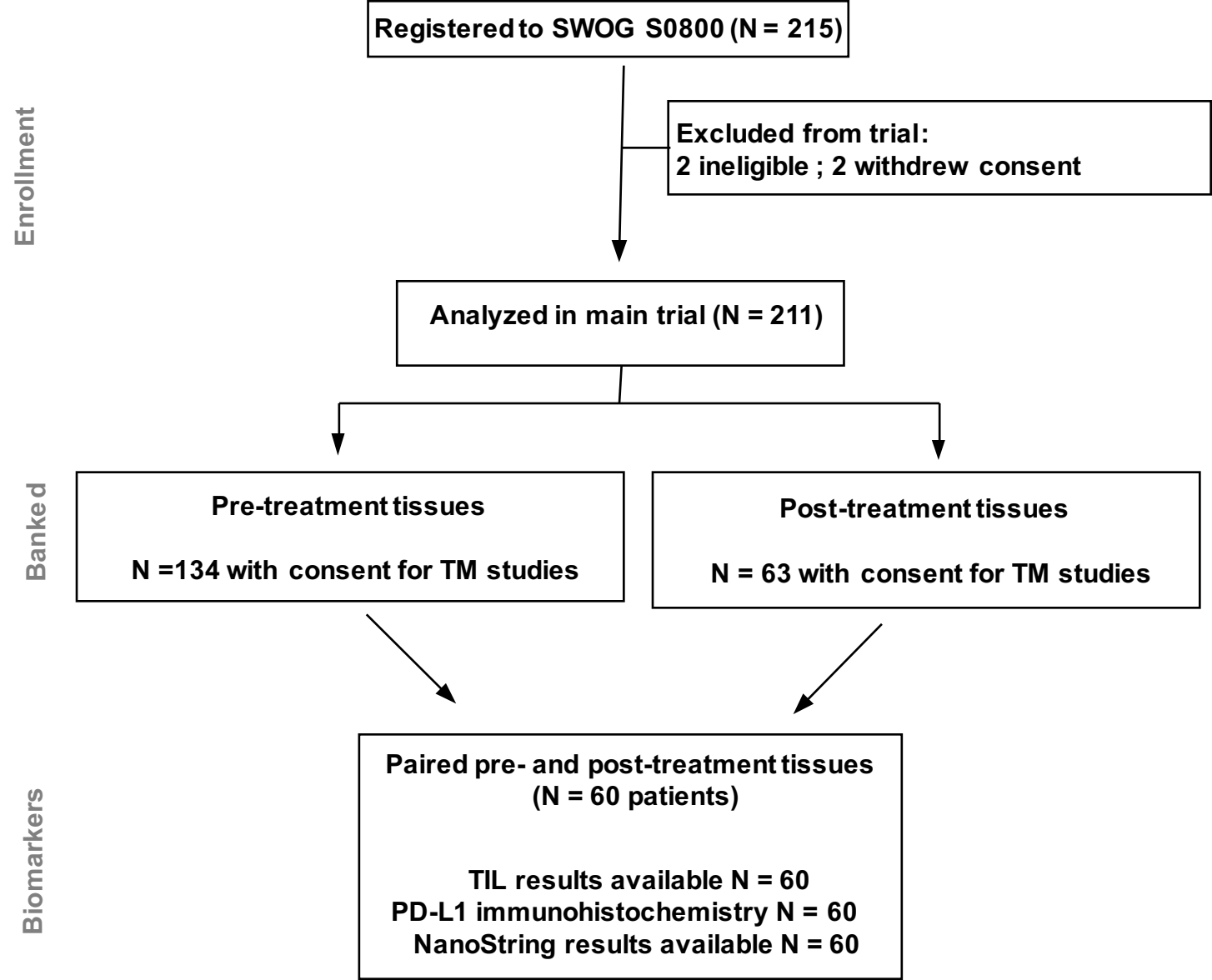

Supplement: Supplementary file 1 — Figure S1. CONSORT diagram of samples used in the study. (PDF 11 kb) [file 40425_2019_563_MOESM1_ESM.pdf]

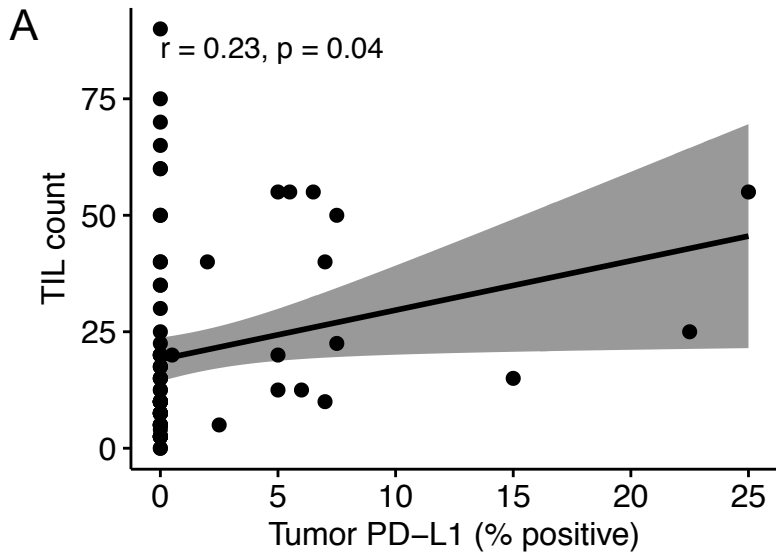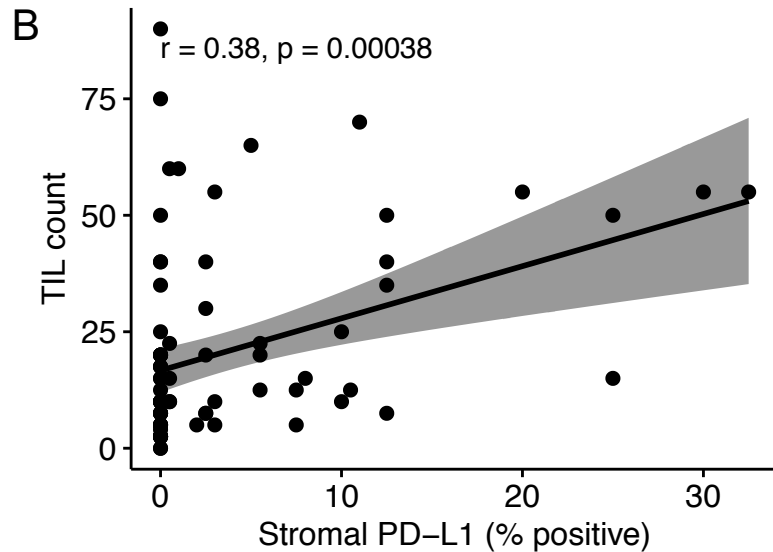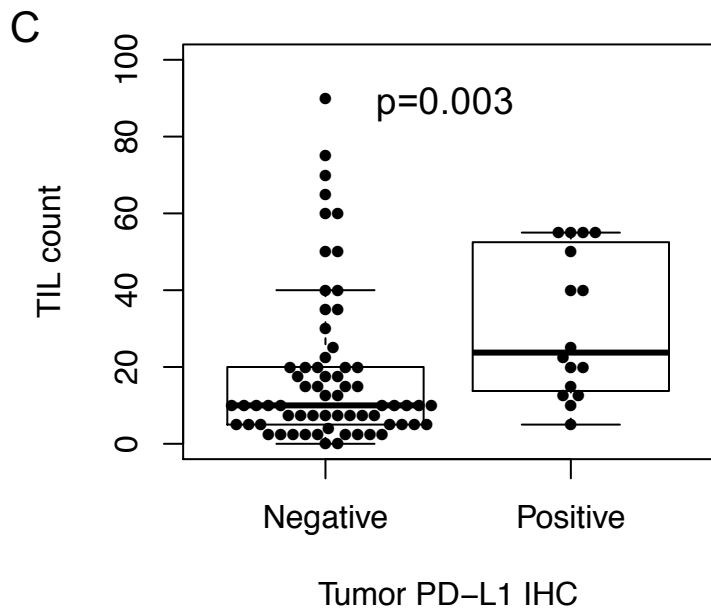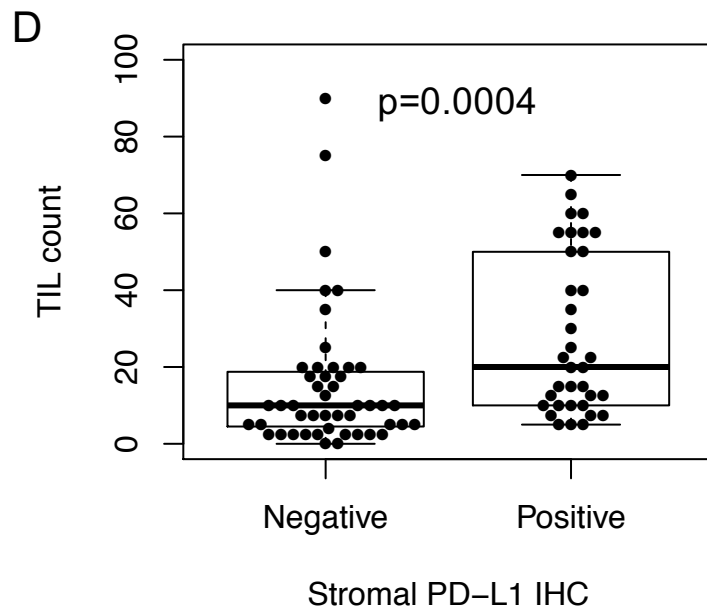

Supplement: Supplementary file 4 — Figure S4. Correlation between TIL counts and PD-L1 expression. A. Correlation between TIL counts and PD-L1 expression on tumor cells. B. Correlation between TIL counts and PD-L1 expression on stromal cells. C. TIL counts in cases with positive and negative PD-L1 expression on tumor cells. D. TIL counts in cases with positive and negative PD-L1 expression on stromal cells. For A and B, Solid line and grey shade represent linear regression line and 95% confidence interval, respectively. Besides, Pearson correlation coefficient r and p-value are added. For C and D, p values are from Wilcoxon test. (PDF 74 kb) [file 40425_2019_563_MOESM4_ESM.pdf]

A

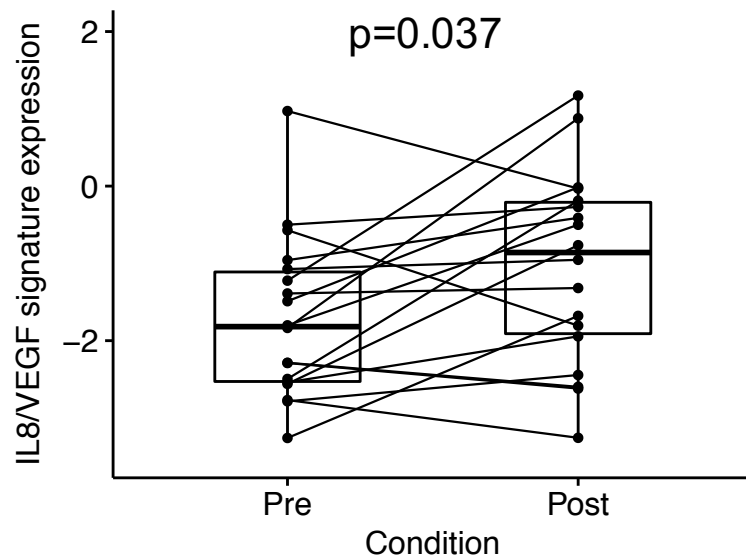

B

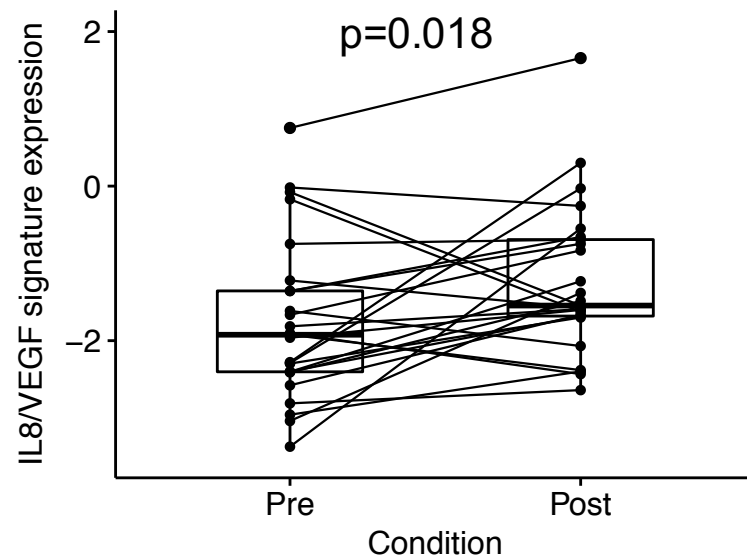

C

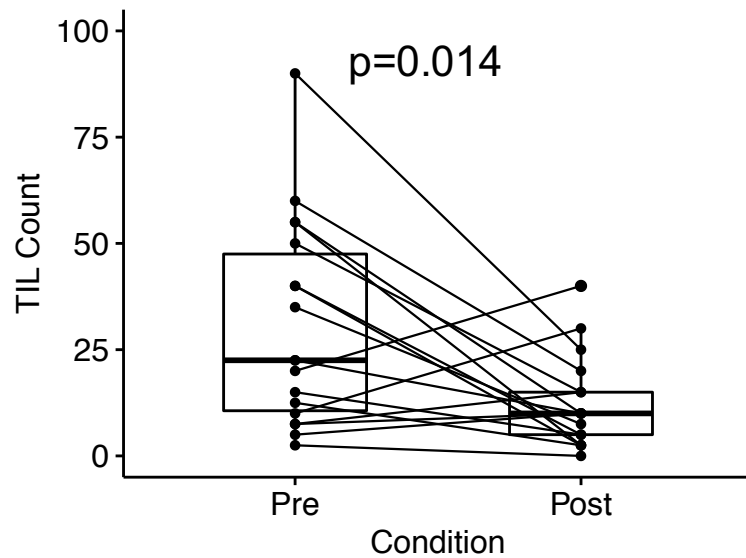

D

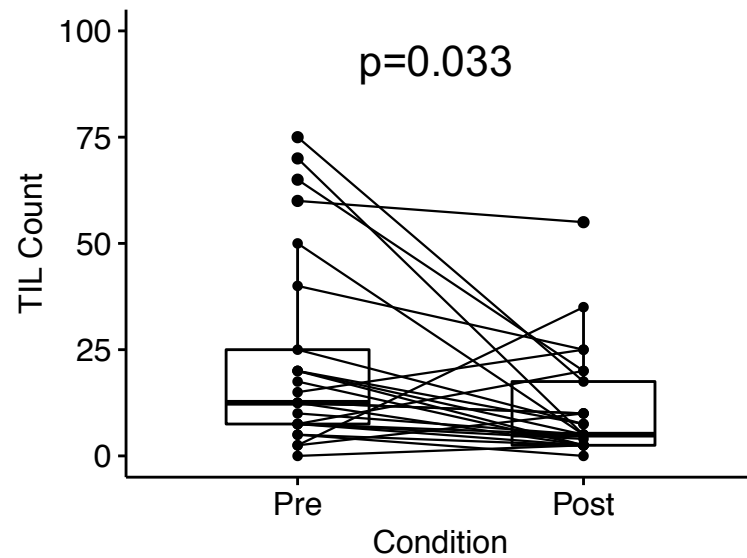

Supplement: Supplementary file 6 — Figure S3. IL8/VEGF signature expression and TIL counts in pre- and post-treatment samples under treatment arms containing or not containing bevacizumab. A. IL8/VEGF signature expression in pre- and post-treatment samples under treatment arm containing bevacizumab. B. IL8/VEGF signature expression in pre- and post-treatment samples under treatment arm not containing bevacizumab. C. TIL counts in pre- and post-treatment samples under treatment arm containing bevacizumab. D. TIL counts in pre- and post-treatment samples under treatment arm not containing bevacizumab. Paired pre- and post-treatment samples are connected by lines to indicate up or down change in each individual. P values are from Wilcoxon test. (PDF 58 kb) [file 40425_2019_563_MOESM6_ESM.pdf]
