# Supplementary material for: Immune profiling of pre- and post-treatment breast cancer tissues from the SWOG S0800 neoadjuvant trial
Source: J Immunother Cancer. 2019 Apr 10;7:88. doi: 10.1186/s40425-019-0563-7 (PMC6457012; doi:10.1186/s40425-019-0563-7)
Supplement: Supplementary file 5 — Figure S2. Age of patient and MKI67 expression in cases with pathologic complete response (pCR) and residual disease (RD). A. Age of patient in cases with pCR and RD. B. MKI67 expressions in all pre-treatment samples with pCR and RD. C. MKI67 expressions in all post-treatment samples with pCR and RD. P values are from Wilcoxon test. (PDF 52 kb) [file 40425_2019_563_MOESM5_ESM.pdf]

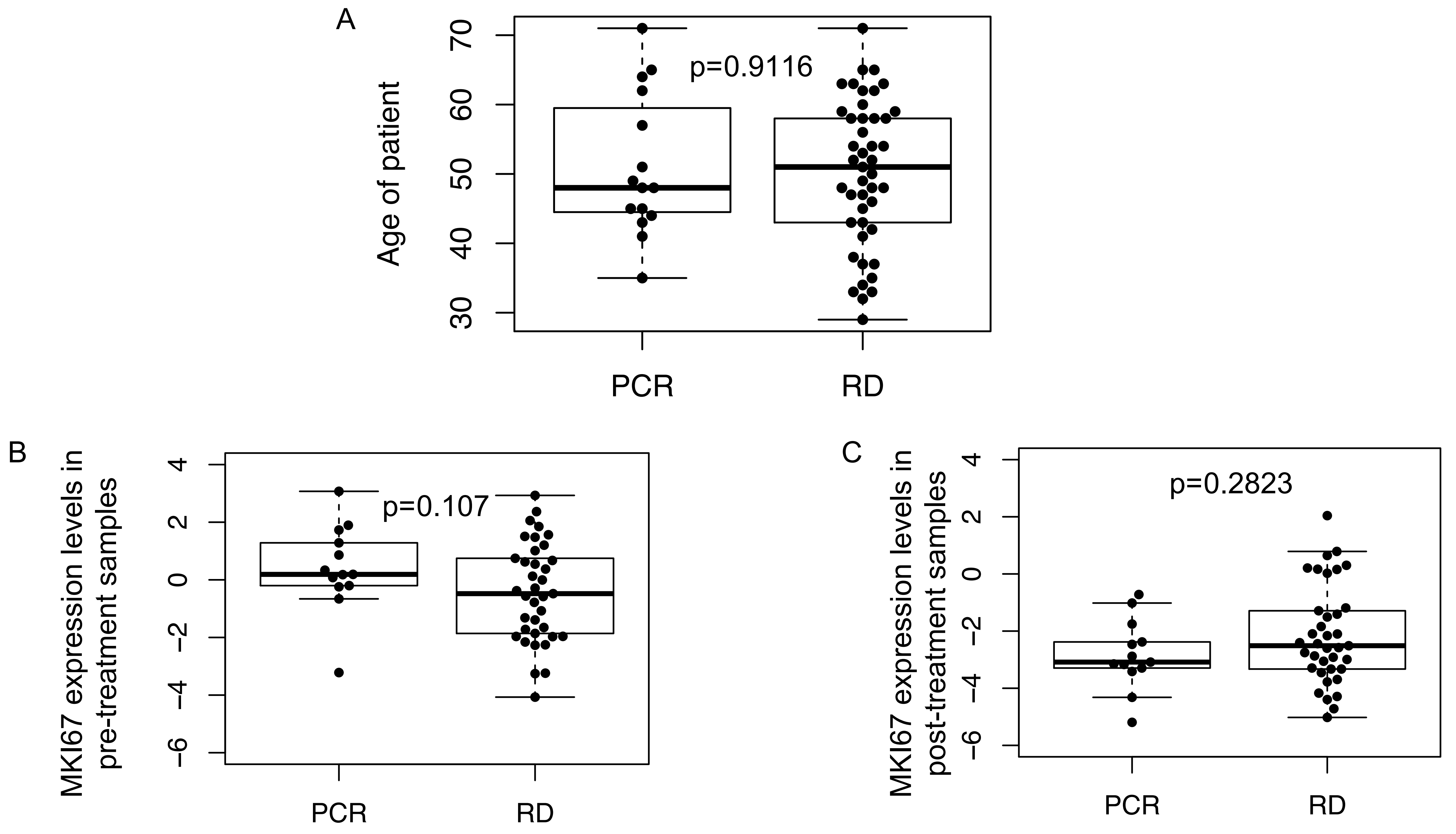

A
Age of patient
70
60
50
40
30
p=0.9116
PCR
RD
B
MKI67 expression levels in pre-treatment samples
4
2
0
−2
−4
−6
p=0.107
PCR
RD
C
MKI67 expression levels in post-treatment samples
4
2
0
−2
−4
−6
p=0.2823
PCR
RD
